# Uræmia Treated by Pilocarpin

**Published:** 1880-07

**Authors:** 


### EXCERPTA.

Uræmia Treated by Pilocarpin.—We learn from *L'Union Medicale* that in the Societe de Biologie, M. Leven communicated the case of a young girl attacked by parenchymatous nephritis induced by a chill, accompanied by œdema of the lower limbs, then by suppression of urine, and finally, on the eleventh day of the disease, by uræmia and a convulsive attack. A hypodermic injection of two centigrammes of nitrate of pilocarpine was given; this produced neither sweating nor salivation. A second injection had equally negative results, but shortly after the third injection there was a redness to the extent of two centimetres around the puncture, then sweating over the body, beginning on the forehead, abundant salivation trickling out of the mouth; after several attempts, vomiting of a yellowish fluid. Half an hour after the injection the patient was passing water under her. The vomited fluid contained traces of ammonia. In the evening there was a fourth injection, followed by sweating and sialorrhœa. 60 grams of saliva contained traces of urea and .098 gram of albumen. 350 grams of urine contained .832 per cent. of albumen. 350 grams of urine contained .842 per cent. of albumen and 2.562 per eent. of urea. On the second day of this treatment (the twelfth of the disease) the patient had recovered her senses, and from this time the amelioration progressed; a fortnight afterward the urine contained no albumen.

